# Editorial: Nutritional quality and nutraceutical properties of Brassicaceae (Cruciferae)

**DOI:** 10.3389/fnut.2023.1292964

**Published:** 2023-10-10

**Authors:** Luke Bell, Maria Jose Oruna-Concha, Antonio De Haro-Bailon

**Affiliations:** ^1^School of Agriculture, Policy & Development, University of Reading, Reading, United Kingdom; ^2^School of Chemistry, Food & Pharmacy, University of Reading, Reading, United Kingdom; ^3^Plant Breeding Department, Spanish National Research Council (CSIC), Madrid, Spain

**Keywords:** glucosinolates, bioavailability, health benefits, cultivation practice, isothiocyanates (ITCs)

The consumption of plant-based foods with health-beneficial properties is one of the crucial factors for the welfare and promotion of health, preventing various chronic conditions like cancer, cardiovascular disease, and neurodegenerative disorders. Plant species belonging to the *Brassicaceae* family are one of the earliest cultivated and domesticated plant groups grown as vegetables, fodder, sources of oils, and for use as condiments. Species from this family are regarded as one of the richest sources of health-promoting phytochemicals, such as minerals, trace elements, polyphenols, vitamins, and isothiocyanates. Many species, such as *Brassica oleracea, Brassica napus*, and *Brassica juncea* are of great economic importance, and include varieties such as broccoli, rapeseed, and mustard. There are many other underutilized species and varieties, which are locally cultivated and vaguely studied, but contribute important components to the human diet (1).

Of the 372 genera and more than 4,000 species of plants belonging to the *Brassicaceae* family, only a limited number of them have been studied and characterized with respect to their composition of compounds of importance to health. What differentiates *Brassica* crops from other horticultural crops is the presence of the secondary metabolites called glucosinolates (GSL), recognized for both their role in plant defense and human health (2). The diversity of compounds derived from glucosinolates, for example, is constantly being expanded. In recent years, numerous new *in planta* metabolic products and cooking-induced derivatives have been identified, and we are only just starting to understand their full significance and contribution toward optimal health.

The study of Coves et al. reports that a mass selection program to modify GSL content was effective in the leaves of two autochthonous *Brassica* species cultivated in northwest of Spain: nabicol (*Brassica napus* L.), selected by glucobrassicanapin (GBN), and nabiza (*Brassica rapa* L.), selected by gluconapin (GNA). However, the response to selection was asymmetrical in both species as it was more effective to decrease selected GSL than to increase it, possibly due to the depletion in the genetic variability necessary to increase the GSL concentration. Interestingly, the selection was also effective in other parts of the plant, suggesting that there is a GSL translocation, or a modification in the compound synthesis pathway that is non-organ specific. This did not have a negative impact on morphological or agronomical characters, suggesting that populations obtained by mass selection may have the same or even better agronomic performance than the original populations.

The article by Montaner et al. studies a traditional winter crop named “bróquil” (*Brassica oleracea* L., var. *italica*) cultivated in Northeast Spain, and locally appreciated for its taste and peculiar flavor. This work evaluates a collection of 13 bróquil landraces, describing their bioactive compounds (total phenolic content, total flavonoid content, total glucosinolate content, and vitamin C), mineral content (macroelements and microelements) and antioxidant activity, and included broccoli as the control. Total phenolic content and total flavonoid content were higher in bróquil landraces than in the broccoli control. In general, bróquil glucosinolate contents, vitamin C, and antioxidant activity were similar to or higher than those in broccoli. All macroelements and microelements showed significant differences among the bróquil accessions and broccoli control, with potassium and iron being the mineral elements detected in the highest amounts. The variability found makes these landraces an important source for the genetic improvement of this crop and contributes to a more diversified and healthy diet.

The consumption of glucosinolates contained in *Brassica* species is correlated with a reduced risk of different types of cancer (3). This anti-cancer effect is mainly associated with certain glucosinolate degradation products, such as isothiocyanates (ITCs), formed during hydrolysis by the enzyme myrosinase (4, 5). The research by Hoffmann et al. concerns assessment of the stability, bioavailability, and bioactivity of two glucosinolate degradation products formed during boiling of red cabbage (*Brassica oleracea* var. *capitata* f. *rubra*). These glucosinolate degradation products are 3-allyl-4-hydroxythiazolidine-2-thione (allyl HTT) derived from sinigrin and 4-hydroxy-3-(4-(methylsulfinyl) butyl) thiazolidine-2-thione (4-MSOB HTT) derived from glucoraphanin. After consumption of boiled cabbage broth, both HTTs can overcome the stomach and intestinal barrier, and passive diffusion seems to be the most likely uptake mechanism. However, no apparent biological effects were observed in the tested assays included in this study, suggesting that HTTs may not contribute to health effects related to GSL consumption. These findings contrast with the properties of other glucosinolate degradation products such as isothiocyanates.

Loose-curd cauliflower is one of the main varieties of plateau summer vegetables in the Lanzhou area (Northwest China). This area is rich in straw resources but the straw recycling rate is low, and straw burning is a common phenomenon. It is in this area of China where the experimental work of Xie et al. is carried out (4). The authors evaluate the impact of straw and plastic film mulching on the yield and quality of open field loose-curd cauliflower (*Brassica oleracea* var. *botrytis* L.). Three experimental mulching methods were studied: dual straw and plastic film mulch (T1), inter-row straw mulch (T2), and full straw mulch (T3), with plots prepared without mulch (CK1) and with plastic film mulch (CK2) as experimental controls. The dry matter accumulation was highest in cauliflower subjected to T1 treatment, followed by those grown under CK2 treatment. In relation to the chemical composition of the harvested products, the work shows that the macro, meso, and micro elements in the loose-curd cauliflower heads grown under T1, T2, and T3 treatments increased in relation to the experimental controls (CK1 and CK2). Moreover, T1, T2, and T3 treatments increased the volatile content, although it remains unclear whether the effect of straw mulching improves the flavor of loose-curd cauliflower. Dual mulching with straw and plastic film appears to be the best application option in corn production areas to produce high-quality, high-yield open field *Brassica* vegetables, as well as crop stalk recycling.

The articles collected in the Research Topic “Nutritional Quality and Nutraceutical Properties of *Brassicaceae (Cruciferae)*” have directly addressed and contributed knowledge in these areas and have advanced the understanding of nutrition and health-related compounds found in *Brassicaceae* species.

## Author contributions

LB: Conceptualization, Writing—original draft, Writing—review and editing. MO-C: Conceptualization, Writing—original draft, Writing—review and editing. AD: Conceptualization, Writing—original draft, Writing—review and editing.
